# Association of neighborhood-level factors with hospitalization for community-associated methicillin-resistant *Staphylococcus aureus*, New York City, 2006: a multilevel observational study

**DOI:** 10.1186/1471-2334-13-84

**Published:** 2013-02-13

**Authors:** Amanda M Farr, Melissa A Marx, Don Weiss, Denis Nash

**Affiliations:** 1 Department of Epidemiology, Mailman School of Public Health, Columbia University, New York, NY, USA; 2 Current affiliation: Custom Data Analytics, Truven Health Analytics, Washington, DC, USA; 3 Bureau of Communicable Disease, New York City Department of Health and Mental Hygiene, New York, NY, USA; 4 Current affiliation: Global AIDS Program, Centers for Disease Control and Prevention, Lukasa, Zambia; 5 Current affiliation: Epidemiology and Biostatistics Program, CUNY School of Public Health at Hunter College, New York, NY, USA

**Keywords:** Antibiotic resistance, Hospitalizations, Multilevel analysis

## Abstract

**Background:**

Hospitalizations with community-associated methicillin-resistant *Staphylococcus aureus* (CA-MRSA) infection have increased in New York City, with substantial geographic variation across neighborhoods. While individual-level risk factors, such as age, sex, HIV infection, and diabetes have been described, the role of neighborhood-level factors (e.g., neighborhood HIV prevalence or income) has not been examined.

**Methods:**

To explore plausible neighborhood-level factors associated with CA-MRSA-related hospitalizations, a retrospective analysis was conducted using New York City hospital discharges from 2006 and New York City-specific survey and health department surveillance data. CA-MRSA-related hospitalizations were identified using diagnosis codes and admission information. Associations were determined by using sex-specific multilevel logistic regression.

**Results:**

The CA-MRSA hospitalization rate varied by more than six-fold across New York City neighborhoods. Females hospitalized with CA-MRSA had more than twice the odds of residing in neighborhoods in the highest quintile of HIV prevalence (adjusted odds ratio [AOR]_Q5 vs. Q1_ 2.3, 95% CI: 1.2, 2.7). Both males and females hospitalized with CA-MRSA had nearly twice the odds of residing in neighborhoods with moderately high proportion of men who have sex with men (MSM) residing in the neighborhood (males: AOR_Q4 vs. Q1_ 1.7, 95% CI: 1.1, 2.7; females: AOR_Q4 vs. Q1_ 2.0, 95% CI: 1.1, 3.6); but this association did not hold for neighborhoods in the highest quintile (males: AOR_Q5 vs. Q1_ 1.2, 95% CI: 0.76, 1.8; females: AOR_Q5 vs. Q1_ 1.5, 95% CI: 0.82, 2.7).

**Conclusions:**

Neighborhood-level characteristics were associated with CA-MRSA hospitalization odds, independent of individual-level risk factors, and may contribute to the population-level burden of CA-MRSA infection.

## Background

Community-associated methicillin-resistant *Staphylococcus aureus* (CA-MRSA) has become a common cause of morbidity in the United States and around the world [1-3]. In 2006, the rate of hospitalization with CA-MRSA in New York City was estimated to be 10.7 per 100,000 people [4], more than twice that of the US population [5], and varied greatly across neighborhoods [4], raising the possibility of neighborhood-level risk factors for CA-MRSA.

Several individual-level factors have been found to be associated with an increased risk of CA-MRSA infection, including male sex [4,5], drug use [6], participation in contact sports [7], sharing of personal items [8,9], and homelessness [6,10]. Neighborhood-level or geographic risk factors are correlated with individual-level factors, but their role in CA-MRSA hospitalization has not been systematically examined. Plausible neighborhood-level risk factors for CA-MRSA include neighborhood HIV prevalence and the proportion of men in the neighborhood who are men who have sex with men (MSM), as rates of CA-MRSA are higher among HIV positive persons [11,12] as well as MSM [13]; neighborhood income distribution, as CA-MRSA risk factors including crowding [14] and limited access to medical care [10] may be more common among persons living in poverty; and levels of emergency department (ED) usage [15], because persons lacking health insurance may rely on the ED for medical treatment for worsening CA-MRSA infections.

In order to evaluate the influence of neighborhood factors on CA-MRSA hospitalizations among New York City residents, we used a combination of data sources and examined the multilevel associations between neighborhood-level factors and individual odds of CA-MRSA hospitalization relative to all other non-*Staphylococcus aureus* hospitalizations, controlling for measured individual-level risk factors. Understanding neighborhood-level risk factors for CA-MRSA, as has been done for a number of other health outcomes [16-18], is important to help to target public health surveillance and interventions for what has become an increasing public health concern.

## Methods

### Study population

We examined data on all hospitalizations except those related to births and those of children under 1 year of age (assuming most were hospitalized for birth in the past year) among New York City residents in 2006. Neighborhood designations were based on aggregations of zip codes into 42 New York City United Hospital Fund (UHF) neighborhoods, ranging in population from approximately 35,000 to 499,000 residents [19].

### Definitions

Data on hospitalizations were drawn from the New York Statewide Planning and Research Cooperative System (SPARCS), an event-based dataset of hospital discharges containing patient demographics, 15 diagnosis fields, billing and address information [20]. Although data were considered identified, they did not contain names, raw birthdates, or street addresses. Patient zip code and UHF neighborhood of residence were used in this analysis.

To identify MRSA hospitalizations, diagnosis fields (principal and other) for each hospitalization in 2006 were evaluated using *International Classification of Diseases*, *Ninth Revision, Clinical Modification* (ICD-9-CM) codes. Hospitalizations with diagnosis codes for *Staphylococcus aureus* pneumonia (482.41), *Staphylococcus aureus* septicemia (038.11), or *Staphylococcus aureus* infection specified elsewhere or unspecified (041.11), and an additional ICD-9-CM (V09.0) indicating resistance to drugs (in this case, methicillin) were considered MRSA-related hospitalizations [4]. MRSA hospitalizations were classified as CA-MRSA if discharge records indicated the patient (1) had MRSA diagnosis on admission based on a present-on-admission indicator; (2) had not been transferred from another health care facility, including nursing homes, long term care facilities and hospitals or was admitted for complications from a surgical procedure; (3) had not been hospitalized earlier in 2006; and (4) was not diagnosed with a chronic condition that results in frequent hospitalization including chronic renal failure, cancer, chronic obstructive pulmonary disease, or congestive heart failure [4,21]. Other MRSA hospitalizations were considered to be healthcare-associated (HA-MRSA).

### Individual-level covariates

Age, sex, recorded diabetes diagnosis, and HIV-related diagnoses were previously found to be associated with CA-MRSA hospitalization in New York City [4] and therefore were included in this analysis. Age was divided into 4 categories: under 18, 18 to 44, 45 to 64, and 65 and greater years of age. Principal or secondary diagnosis codes, based on ICD-9-CM codes, were used to classify hospitalizations related to diabetes and a pre-existing indicator based on ICD-9-CM codes and disease-related groupings (DRGs) was used to identify HIV.

### Neighborhood-level variables

HIV prevalence, the percentage of UHF neighborhood residents living with HIV/AIDS, was estimated using the number of persons diagnosed and living with HIV/AIDS by UHF neighborhood provided by the Bureau of HIV/AIDS at the New York City Department of Health and Mental Hygiene (NYCDOHMH) [22] and New York City UHF neighborhood population estimates [23]. MSM proportion, defined as the proportion of men who report having sex with at least one man in the past year, was based on survey data collected by the New York City Community Health Survey, an annual random-digit-dial survey of 10,000 residents [24]. Aggregate estimates combining survey data from 2003 to 2006 were created by the Bureau of Epidemiology Services at NYCDOHMH. Data on neighborhood income came from the 2000 US Census. Median household income for each zip code was averaged to create UHF neighborhood estimates. The number of emergency department visits by neighborhood of residence [25] was provided by the Syndromic Surveillance Unit at the NYCDOHMH and were converted to UHF neighborhood rates (ED visits per 100,000 population) based on New York City population estimates [23]. UHF neighborhood-level variables were divided into quintiles for the purposes of statistical analyses.

### Analysis

To examine differences in CA-MRSA rates across neighborhoods, we mapped the 2006 CA-MRSA hospitalization rates by UHF neighborhood population. Mapping was performed with ArcGIS 9. To determine the associations between neighborhood-level characteristics and hospitaliza-tion with CA-MRSA at the individual-level, we employed multilevel modeling to compare characteristics of persons hospitalized with CA-MRA were compared to those of non-*Staphylococcus* hospitalizations. Multilevel modeling, also known as hierarchical modeling, was necessary because the data were organized at the neighborhood- and individual-levels; the individuals were nested within neighborhoods. We hypothesized neighborhood-level factors would be associated with CA-MRSA hospitalization independent of measured individual-level risk factors. To identify individual-level factors associated with CA-MRSA hospitalization, we constructed sex-specific logistic regression models with only individual-level factors. We then constructed similar models including a single neighborhood level factor. Finally, multilevel models that contained both individual-level factors and a neighborhood-level factor were constructed to explore sex-specific associations between neighborhood-level factors and CA-MRSA hospitalizations, adjusting for individual-level covariates. We report crude and adjusted odds ratios with 95% confidence intervals. To reduce the impact of possible misclassification, HA-MRSA hospitalizations and hospitalizations with any other *Staphylococcus* diagnoses were excluded from the analysis. Analyses were performed with SAS 9.1.

SPARCS hospitalization data was obtained through a data use agreement between the NYCDOHMH and SPARCS Data Protection Review Board. This project was approved by the NYCDOHMH Institutional Review Board as research involving materials that have already been collected.

## Results

### Descriptive and crude analyses

**Figure 1:**
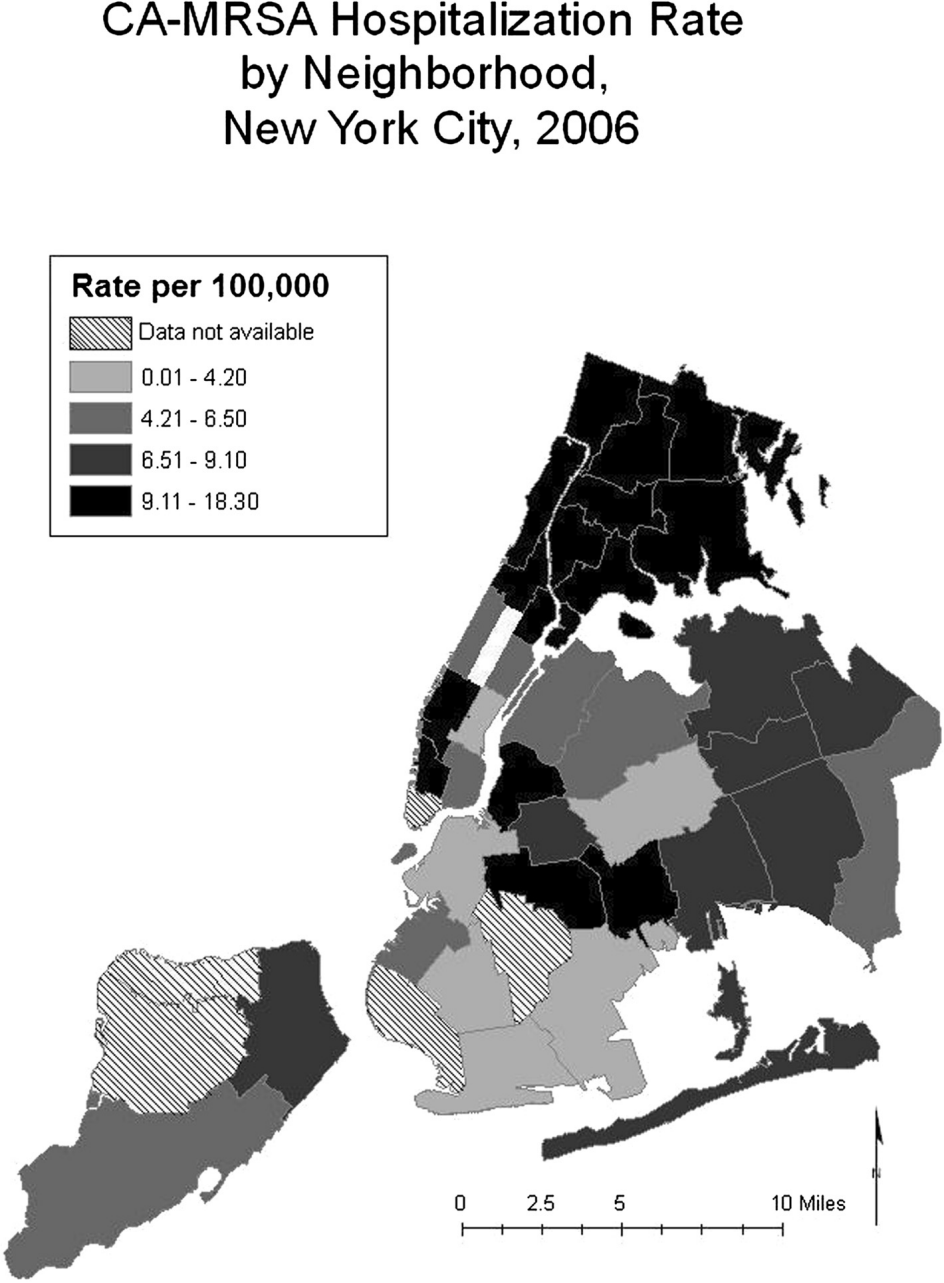
CA-MRSA hospitalization rate per 100,000 people, New York City neighborhoods, 2006.

Among New York City residents in 2006, there were 645 CA-MRSA hospitalizations and 908,184 non-*Staphylococcus* hospitalizations. The CA-MRSA hospitalization rate varied by more than six-fold across neighborhoods, ranging from 3.0 to 18.3 per 100,000. Higher rates clustered in certain areas, including the Bronx and some UHF neighborhoods in Manhattan (Figure 1).

Sex-specific bivariate analysis of crude associations between individual-level age, diabetes diagnosis, and HIV diagnosis are shown in Table 1. Among males, those hospitalized with CA-MRSA tended to be younger, with an odds ratio (OR) of 3.5 (95% CI 2.3, 5.2) for those under 18, 4.0 (95% CI 3.0, 5.4) for those aged 18 to 44, and 2.4 (95% CI 1.8, 3.3) for those aged 45 to 64, each compared to 65 and older. Among females, CA-MRSA hospitalizations were as likely to be 18 to 44 years old as they were to be 65 and older (OR 1.1, 95% CI 0.75, 5.5). CA-MRSA hospitalizations had more than 3 times the odds of having an HIV diagnosis (males: OR 3.3, 95% CI 2.5, 4.3; females: OR 3.1, 95% CI: 1.9, 5.1). Having a diabetes diagnosis was significantly associated with CA-MRSA hospitalization only among females (OR 1.4, 95% CI 1.1, 1.9).

**Table 1:** Crude odds ratios for associations between individual-level and neighborhood-level characteristics and hospitalizations with CA-MRSA, New York City, 2006

	**Males**		**Females**	
	**OR**	**95% CI**	**OR**	**95% CI**
***Individual***				
**Age (years)**				
< 18	3.5	2.3, 5.2	4.7	3.1, 6.9
18-44	4.0	3.0, 5.4	1.1	0.75, 1.5
45-64	2.4	1.8, 3.3	1.9	1.4, 2.6
≥ 65	1.0	-	1.0	-
**Diabetes**				
Yes	0.91	0.72, 1.2	1.4	1.1, 1.9
No	1.0	-	1.0	-
**HIV**				
Yes	3.3	2.5, 4.3	3.1	1.9, 5.1
No	1.0	-	1.0	-
***Neighborhood***				
**HIV Prevalence**				
≤ 0.37%	1.0	-	1.0	-
0.38-0.73%	1.2	0.75, 2.0	1.6	0.90, 2.9
0.74%-1.3%	1.6	0.97, 2.7	1.9	1.0, 3.4
1.3-1.65%	1.6	0.98, 2.7	1.2	0.65, 2.4
> 1.65%	1.7	1.0, 2.7	2.7	1.5, 5.0
**MSM Proportion**				
≤ 5.7%	1.0	-	1.0	-
5.8-7.0%	1.4	0.81, 2.3	2.0	1.1, 3.8
7.1-8.0%	1.6	1.0, 2.4	1.6	0.94, 2.8
8.1-9.8%	1.9	1.2, 3.1	2.1	1.1, 3.9
> 9.8%	1.4	0.90, 2.2	1.5	0.84, 2.8
**Income per year ($)**				
≤ 26,000	1.2	0.82, 1.9	1.2	0.70, 2.1
26,001-35,000	0.81	0.51, 1.3	0.64	0.35, 1.2
35,000-41,000	1.30	0.85, 2.1	0.97	0.52, 1.8
41,000-48,000	0.69	0.44, 1.1	0.77	0.44, 1.4
> 48,000	1.0	-	1.0	-
ED Usage Rate*				
≤ 24,000	1.0	-	1.0	-
24,001-32,600	0.68	0.42, 1.1	1.0	0.54, 2.0
32,601-44,200	1.1	0.71, 1.6	1.4	0.76, 2.4
44,201-56,000	1.3	0.86, 2.0	1.4	0.77, 2.6
> 56,000	1.2	0.78, 1.8	1.8	0.99, 3.3

NOTE. CI, confidence interval; ED, emergency department; HIV, human immunodeficiency virus; MSM, men who have sex with men; OR, odds ratio.

* Emergency department visits per 100,000 people.

In crude sex-specific analyses including only neighborhood HIV prevalence, those hospitalized with CA-MRSA were significantly more likely to come from the highest HIV prevalence neighborhoods in NYC compared with those from the lowest prevalence neighborhoods for both males (OR_Q5 vs. Q1_ 1.7, 95% CI 1.0, 2.7) and females (OR _Q5 vs. Q1_ 2.7, 95% CI 1.5-5.0). Males and females hospitalized with CA-MRSA were also more likely to be from neighborhoods with moderately high versus lower MSM proportion (males: OR _Q4 vs. Q1_ 1.9, 95% CI 1.2, 3.1; females: OR _Q4 vs. Q1_ 2.1, 95% CI 1.1, 3.9), but the association did not hold at higher levels of neighborhood MSM proportion. There was no significant crude association with neighborhood income distribution or ED usage (Table 1).

### Multivariable analysis

In multivariable logistic regression models, all individual-level risk factors examined were statistically significantly associated with CA-MRSA hospitalization. Individual-level risk factors remained significant and of similar magnitude with the inclusion of each neighborhood-level characteristic in separate sex-specific multilevel models except diabetes, which was not significant in the crude model for hospitalized males but was significant in all adjusted models (adjusted odds ratio (AOR) 1.3, 95% CI 1.0, 1.7) that included a neighborhood factor.

After adjusting for individual-level risk factors, females hospitalized with CA-MRSA had more than twice the odds of residing in UHF neighborhoods with the highest HIV prevalence compared to the lowest (AOR_Q4_ vs. Q1 2.3, 95% CI 1.2, 2.7). Though adjusted odds ratios at other levels of HIV prevalence were not statistically significant, the odds of hospitalization with CA-MRSA increased with increasing HIV prevalence. There were no significant differences by neighborhood HIV prevalence among males. In separate models, both males and females hospitalized with CA-MRSA had nearly twice the odds of residing in neighborhoods with moderately high proportion of men who have sex with men residing in the neighborhood (males: AOR_Q4 vs. Q1_ 1.7, 95% CI: 1.1, 2.7; females: AOR_Q4 vs. Q1_ 2.0, 95% CI: 1.1-3.6); but this association did not hold for neighborhoods in the highest quintile proportion of MSM (males: AOR_Q5 vs. Q1_ 1.2, 95% CI: 0.76, 1.8; females: AOR_Q5 vs. Q1_ 1.5, 95% CI: 0.82-2.7). UHF neighborhood income and ED usage rate were not significant predictors of CA-MRSA hospitalization in multilevel analyses for males or females (Table 2).

**Table 2:** Adjusted odds ratios for associations between neighborhood-level characteristics and hospitalizations with CA-MRSA, controlling for individual-level characteristics, New York City, 2006

	**Males**		**Females**	
	**AOR**	**95% CI**	**AOR**	**95% CI**
**HIV Prevalence**				
≤ 0.37%	1.0	-	1.0	-
0.38-0.73%	1.1	0.67, 1.7	1.5	0.84, 2.7
0.74%-1.3%	1.3	0.81, 2.2	1.7	0.90, 3.1
1.3-1.65%	1.2	0.73, 2.0	1.1	0.56, 2.1
> 1.65%	1.1	0.71, 1.9	2.3	1.2, 2.7
**MSM Proportion**				
≤ 5.7%	1.0	-	1.0	-
5.8-7.0%	1.2	0.72, 1.9	1.9	1.0, 3.5
7.1-8.0%	1.3	0.86, 1.9	1.4	0.84, 2.5
8.1-9.8%	1.7	1.1, 2.7	2.0	1.1, 3.6
> 9.8%	1.2	0.76, 1.8	1.5	0.82, 2.7
**Income per year ($)**				
≤ 26,000	0.94	0.64, 1.4	0.95	0.55, 1.6
26,001-35,000	0.76	0.49, 1.2	0.58	0.32, 1.1
35,000-41,000	1.3	0.85, 1.9	0.89	0.49, 1.6
41,000-48,000	0.69	0.45, 1.0	0.72	0.41, 1.3
> 48,000	1.0	-	1.0	-
ED Usage Rate*				
≤ 24,000	1.0	-	1.0	-
24,001-32,600	0.63	0.40, 1.0	0.98	0.51, 1.9
32,601-44,200	0.92	0.62, 1.4	1.2	0.69, 2.2
44,201-56,000	1.0	0.70, 1.6	1.2	0.64, 2.2
> 56,000	0.83	0.55, 1.3	1.4	0.76, 2.6

NOTE. AOR, adjusted odds ratio; CI, confidence interval; ED, emergency department; HIV, human immunodeficiency virus; MSM, men who have sex with men.

*Emergency department visits per 100,000 people.

## Discussion

Our analyses suggest important, biologically plausible neighborhood-level factors that may, in part, drive CA-MRSA hospitalization rates at the population-level in New York City. In sex-specific multilevel analyses, strong positive associations between higher UHF neighborhood HIV prevalence and MSM proportion with higher odds of CA-MRSA hospitalization persisted, even after controlling for individual factors. Additionally, important differences in individual and neighborhood-level risk factors for CA-MRSA hospitalization between males and females were observed.

Our analysis showed that neighborhood HIV prevalence was associated with increased odds of CA-MRSA hospitalization among females even after controlling for individual HIV-status. While the associations between HIV prevalence and odds of hospitalization with CA-MRSA were not statistically significant at lower HIV prevalence levels, a dose–response relationship is suggested. Studies, including ours, have found HIV positive persons to be at an increased risk for CA-MRSA infection and hospitalization with CA-MRSA, possible due to increased viral load, weakened immune systems, lack of antiretroviral therapy, or problems with skin integrity [4,11,26,27]. This raises the possibility that neighborhood HIV prevalence may play a role in CA-MRSA transmission even among HIV-negative persons, and that the attributable fraction of CA-MRSA hospitalizations associated with HIV may be greater than previously thought.

We also found higher neighborhood MSM proportion to be associated with CA-MRSA hospitalization for both males and females. While we did observe differences by neighborhood MSM proportion at the extreme ends of the distribution, we did not observe a dose–response relationship and the association did not extend to neighborhoods in the highest quintile of neighborhood MSM proportion. A population-based study in San Francisco found that zip codes with higher percentage of partnerships being same-sex male also had higher rates of CA-MRSA infection [28]. In New York City-based analyses, high rates of CA-MRSA observed among MSM were associated with HIV infection, crystal methamphetamine use, physical contact with someone with a skin infection, sex at private parties, and perhaps membership in social networks that include others engaged in these behaviors [13]. Though we were unable to control for MSM status at the individual-level in our analysis of males, the finding that neighborhood proportion of MSM was associated with the risk of CA-MRSA hospitalization among females is intriguing, and suggests the possibility of a neighborhood-level effect. Epidemiologic data suggest that HIV prevalence among MSM in New York City is high (e.g., 8.8% overall, and 17.7% among MSM in Manhattan) [29], making it difficult to distinguish between the potentially independent role of each factor. The lack of a dose–response relationship among males or females, however, make these results difficult to interpret, and the association between neighborhood MSM proportion and CA-MRSA needs to be evaluated in future research before stronger conclusions can be drawn.

Epidemiologic studies have shown that neighborhood factors have independent associations with a number of health outcomes, including neighborhood socioeconomic status with diabetes [18] and heart disease [16], and neighborhood poverty with relapse into injecting drug use [17]. Although neighborhood characteristics have commonly been explored with regard to chronic diseases and health outcomes, they likely also affect infectious disease risk. The extent of transmission or acquisition in a given geographic area is dependent upon a number of relevant factors that may in fact vary by neighborhood, including the number of hosts susceptible to infection and the number of hosts who are currently infected or are carriers of the microbial agent. Contacts between infectious and susceptible persons will occur in many settings, including the neighborhood in which infectious persons reside [30].

Our analysis has limitations that merit discussion. At the individual level, V-code 09.0 and ICD-9 CM codes indicating *Staphylococcus* infection have been used in published literature to identify MRSA cases in administrative data [4,31-34] though some analyses have shown conflicting results as to the accuracy of these definitions [35,36]. Schweizer, et al. found that use of administrative coding lacked sensitivity and have low positive predictive value for hospital-associated infections [35]. A second study by Schaefer, et al. found low sensitivity, leading to an underestimation of the number of cases, but high positive predictive value particularly for community-associated infections [36]. In addition, lack of laboratory data and healthcare history may have led to misclassification of susceptible versus resistant infection and of community- versus hospital-associated infection.

It was not possible to apply the Centers for Disease Control and Prevention’s definition of CA-MRSA to our dataset [37]; however, study inclusion and exclusion criteria were used as a proxy. Patients could not be linked across calendar years and therefore examining the full 12 months prior to hospitalization was not possible. This particularly affects those patients hospitalized early in 2006. Additionally, the definition used in this analysis likely resulted in exclusion of some CA-MRSA cases (those with certain chronic diseases also associated with HA-MRSA) but was necessary to improve the specificity of our outcome definition. *Staphylococcus* infections which were not recorded on the hospital discharge record or incorrect diagnosis coding could have resulted in some misclassification. Specifically, *Staphylococcus* infections other than CA-MRSA may have been incorrectly classified as CA-MRSA rather than excluded from analysis. Finally, the primary cause of hospitalization need not have been CA-MRSA, which may lead to an overestimation of hospitalizations primarily due to CA-MRSA.

At the UHF neighborhood level, HIV prevalence, MSM proportion, and ED usage values may be underestimates due to lack of diagnosis, reporting, or inaccurate survey responses and 2000 Census data may not accurately represent neighborhood income in 2006. Misclassification of these neighborhood-level covariates was non-differential by outcome status at the individual-level. Additionally, neighborhood measures are aggregations of individual-level information. Variation will exist among individuals living in a single neighborhood. Finally, there are many ways to designate “neighborhoods” in geographic analyses, including UHF, zip code, cities, et cetera and the specific designations used in our analysis may have affected our results. Due to small numbers of neighborhoods, we were unable to control for multiple neighborhood-level factors in the same model, 7which could result in uncontrolled confounding at the neighborhood level. We were also unable to measure individual socioeconomic status and MSM status and therefore could not control for them in modeling.

## Conclusions

CA-MRSA-associated hospitalizations are relatively common in New York City and elsewhere, with substantial geographic variability. Using hospital discharge data, we were able to examine nearly 1 million hospitalizations in a population-based analysis, providing an extensive view of the New York City population. Our combination of this data with other population-level sources available gave us a unique opportunity to examine possible neighborhood-level risk factors for CA-MRSA hospitalizations while controlling for important individual risk factors. This analysis suggests that, over and above individual-level risk factors, CA-MRSA hospitalization may be linked to neighborhood-level characteristics such as HIV prevalence and MSM proportion. It is important to further understand the epidemiology of this infectious disease in order to initiate prevention strategies targeted to appropriate populations and risk factors. As many public health interventions are undertaken at the neighborhood-level, it is essential to elucidate possible neighborhood-level risk factors for morbidity. Future research should focus on examining the impact of neighborhood-level factors such as HIV prevalence and MSM proportion on the incidence of CA-MRSA and *Staphylococcus aureus* infections, as well as hospitalizations in other cities and nationally (i.e., over wider geographic areas) and to further determine the independent role of each of these factors. Additionally, more focused research studies are needed to understand the complex nature of the high CA-MRSA hospitalization rates found in this study in certain neighborhoods of New York City, such as the Bronx.

## Abbreviations

AIDS: Autoimmune deficiency syndrome; AOR: Adjusted odds ratio; CA-MRSA: Community-associated methicillin-resistant *Staphylocccus aureus*; ED: Emergency department; HA-MRSA: Healthcare-associated methicillin-resistant *Staphylococcus aureus*; HIV: Human immunodeficiency virus; ICD-9-CM: International classification of diseases: ninth edition: clinical modification; MRSA: Methicillin-resistant *Staphylococcus aureus*; MSM: Men who have sex with men; NYCDOHMH: New York City Department of Health and Mental Hygiene; OR: Odds ratio; SPARCS: Statewide planning and research cooperative system; UHF: United hospital fund

## Competing interests

The authors declare that they have no competing interests.

## Authors’ contributions

AMF, MAM, DW, and DN conceived and designed the study. MAM and DW aided in acquiring study datasets. AMF performed statistical analyses. AMF and DN interpreted results. AMF wrote the report. MAM, DW, and DN commented and revised report. All authors read and improved final manuscript.

## Pre-publication history

The pre-publication history for this paper can be accessed here:

http://www.biomedcentral.com/1471-2334/13/84/prepub
